# Medicine Not an Exact Science

**Published:** 1868-04

**Authors:** J. W. H. Baker

**Affiliations:** President of the Iowa State Medical Society


### MEDICINE NOT AN EXACT SCIENCE.

ADDRESS BY J. W. H. BAKER, M. D.,

President of the Iowa State Medical Society, delivered before the Society at its session in Davenport, May 22d, 1867.

*Gentlemen of the Iowa State Medical Society:*

But a few years since one of the best medical authors, in the preface to his newly published work on medicine, announced the fact that the Science of Medicine had already taken her position as one of the exact sciences. Still later a member of our profession, in reading an essay, announces that "Medicine *is not* one of the exact sciences, and the attempts that have been made to place it in that rank have sufficiently shown its imperfections. There are too many elements at work, and we must observe their operations, but not attempt to draw the line of action for them too closely. We must be content, then, to take medicine as it is. It has made a great advance towards exactitude of late years."

The assertion that we must take medicine as a science as it is, is no more true now than it was half a century ago; but the advances being great, we can make it our duty to add to those advances. Many of our professional brethren may contend that Medical Science is advancing in its progress, while others may believe this to be otherwise. In the treatment of disease nothing is more marked than the change in the treatment of acute diseases.

Pneumonia less than a quarter of a century ago was treated generally by blood-letting, and now by many physicians with extra nutrients and stimulation, while a few rely on hygienic restrictions without medicine, and assert that the results of such practice is equal in efficacy to any other known method.

Gastro Enteric Fever, but a few years since, was, and by many at the present day is, treated actively, with the belief that a marked, reliable influence is obtained by the active use of powerful remedies; while most of the profession are in doubt as to the efficiency of remedies in curing such disease.

Many other similar facts might be cited to show the want of exactitude in practical medicine. From this very want of exact science the profession of medicine is made the arena for dissenting professional opinions and disagreement, which, among many of our patrons, causes a want of confidence in the members of the regular profession and the introduction to lucrative practice of all the quackish men and arts with which we as a people are burdened. The treatment of disease is either empirical or rational: although by the former we may do much good, still the true or rational method should be adopted as far as possible. "The difference between the scientific practitioner and the empiric, regular or irregular, is never better seen than in the treatment of incurable disease; where one desists from all active measures that have not some definite object, the other harasses his patient to the last with worse than useless experiments."

While medicine has two great principal objects in view: first, the knowledge of diseases; and second, their cure, we are obliged to admit that under the curative relation, as the second object, few diseases are submitted to the Empire of Medicine: that is, subject to the full and positive control of medicinal agents. Physicians standing high in the arena of medical life, and writing for the medical public; other physicians writing for a more literary and general public purpose, are frequently reminding us of the imperfection of our great science. Holmes, one of the most profound of our Anatomical Professors, during the last quarter of a century, says, in an address to the State Medical Society of Massachusetts, as follows: "Throw out Opium, which the Creator himself seems to prescribe; for we often see the scarlet poppy growing in the cornfield, as if it were foreseen that wherever there is hunger to be fed, there must also be pain to be soothed. Throw out a few specifics which our art did not discover, and is hardly needed to apply; throw out wine, which is food, and the vapors which produce the miracle of anesthesia, and I firmly believe that if the whole materia medica, *as now used*, could be sunk to the bottom of the sea, it would be all the better for mankind, and all the worse for the fishes."

Holland, a literary writer of no small calibre, says to the regular profession, in his Letters to the Jones's: "Homœopathy is to do you and your friends good in spite of yourselves. It has already modified your practice, while you have been talking and acting against it. You are not exhibiting to-day a third as much medicine as you did before Homœopathy made its appearance." These are not isolated cases in which our professional brethren force upon us a necessary belief in the imperfection of our science. Forbes, and Bigelow, and many other writers and believers in rational medicine, are united in the effort to establish a more perfect reliance on nature as the great restorer. There is an acknowledged natural power existing in the system to overcome disease, and we cannot deny such fact while we remain reasonable practitioners searching for truth.

It is fast becoming a determined fact that many of the acute diseases from which mankind suffer will terminate within a generally limited space of time for recovery without the effect of positive action of medicine, and being convinced of this fact, we are necessarily led to endorse the common assertion that as a physician grows older in practice, the less number of remedies he relies upon as remedial medical agents. The great expectations of many a young practitioner of medicine are sadly spoiled when his special reliance is placed on the effect of medicine alone; or, on the contrary, while warring with disease, how often does nature with her own kind hand give him opportunity to win laurels of praise for his most unmerited skill? Bigelow, in his Essay on Self-limited Diseases, says: "The young student goes forth into the world believing if he does not cure disease it is his own fault; yet when a score or two of years have passed over his head, he will come at length to the conviction that some diseases are controled by nature alone." I have known a young practitioner of the healing art, after having applied remedies day after day for the purpose of restoring a diseased child, at last abandon the case as hopeless, and recommend a cessation of medicinal measures, as they had proved useless, and within a few hours the abandoned case came through a natural crisis to assume a power to recover, and thereafter convalescence continue to perfect health.

I have known a Medical Professor, of marked ability and accustomed research, who had been busily engaged in the practice of his profession for nearly a quarter of a century, and had learned in his student life that venesection in acute diseases was the only effective remedy, and therefore had practiced upon that instruction in his treatment of Pneumonia. This professor of medical art, after a due deliberation, and under a cloud of doubt as it were, came to the very daring conclusion to try the effect of treatment in Pneumonia without blood-letting, and made to himself the astounding discovery, and openly asserted that in ten days' time his patients treated without bleeding were very much farther advanced toward convalescence than those of his associates who were practicing after the former plan. Not that he relied on nature, but that he modified his method and made a less severe attack upon his antagonist.

Still more recently I read in one of our best medical journals a question of considerable importance in relation to Rheumatism. The question is: "How long does Rheumatism take to get well itself?" "Until that point is settled, how could they determine what to do?" "Who could venture to say this or that treatment was or was not successful, if he did not know how long the disease was to last of itself?" After some reasons pro and con this questioner says his view was that acute rheumatism cures itself. "It had once been said that six weeks in bed was the best cure for everything, and Dr. Stewart's rheumatic cases mentioned had an average of forty-two days, which was just six weeks;" and to these instances the six weeks in bed cure was evidently well adapted. If the science of medicine were an exact science, we should never be able to record such changes, such variable practices, or such universal success in the use and disuse of medicinal agents. Our profession is one of those called *noble*, but the title is not always significant of the morale of those who practice under it. To be sure we have the record of many noble examples of worthy and high-minded men in our profession; men born, as it were, to exalt, to ennoble, to advance the standard of our calling; men who reverence the truth, and were never known to equivocate, and for these good qualities were esteemed the very Washingtons of our profession. While on the other hand we are obliged to admit that some of the most flagrant crimes of the present day are those connected with the practice of practitioners of medicine. Record shows that we are professionally linked with men who have no reverence for truth, and who will equivocate, and more than this, will, for pecuniary gain, consent to destroy life. We can but question such. Will the standard of medicine be advanced, its interests promoted, and the social position of a physician sustained, who, for the sum of fifteen or twenty dollars, contracts to procure an abortion? Will the labors of a life-time spent in the Professor's garb, and at last culminating in the murder of a creditor, ever bleach itself from the dark spots of our professional crimes? Much has been written upon the subject of criminal abortion, and much more needs to be written, in order to correct the public opinion that there is no crime in inducing this act. If it is not a crime, why do all legal codes represent enactments for the punishment of it as a crime? Physiological investigation represent it as a thing of life from the moment of conception, and Scripture pronounces a curse upon the man who should commit a much smaller crime, (who should spill his seed upon the ground.) If such are cursed, how much more is he to be cursed who unnecessarily and knowingly causes the destruction of the vitalized ova?

The public look upon the regular practitioner of medicine, whose starting points for medical education, and whose research among the things of antiquity, have given him the practical experience of ages, as belonging to the Old School. The term Old School is very indefinite. What do they mean by Old School? Do they mean that its members cherish a method of practice which has been handed down for a thousand years without improvement? Do they mean that this body of men hold a perfect plan susceptible of no improvement? If these constitute their opinion of the Old School, then the opinion is false. But if by the term Old School they include those who receive thorough instructions from well established sources, in relation to that which is both ancient and modern in medicine, those who practice after a rational method, those who administer whatever has been found useful in disease, and those by whom many of the most important discoveries in medicine have received a practical application; in fine, if they mean the scientific, thoroughly educated, progressive body of men who form the teachers in our Medical Schools, and the practitioners who are not tied to some exclusive dogma, or who do not strike their colors and turn themselves and their conscience over for popular favor or pecuniary gain, then it is an honor to belong to the Old School.

I cannot well pass over in my few words addressed to you, my professional brethren, some of the outgrowths or off-shoots from Medical Science. In the first place I will merely refer to a subject introduced by resolution before our recently assembled American Medical Association. This resolution asked this Association of the representatives of the medical profession of our nation to acknowledge and fellowship the educated female practitioners. This was no strange step in the progress of medicine, when we are informed that such men as ths eminent *Brooklyn divine* urges as an advance in political civilization that *all women* be admitted the privilege of suffrage and an eligibility to political offices. There are reasons why such might be admissible, but many other reasons exist why such rights should not be granted. In this instance the resolution was tabled, and the women left, as Titcomb says, to do as they please. To quote a few lines illustrative of my feeling, as well as that of the author quoted, might not be inappropriate in this place. "There is a weak place, or a wrong place, or a rotten place in the character of every woman who stands and howls upon the spot where her Creator placed her, and neglects her own true work and life while claiming the right to do the work and live the life of man. I will admit all the right that *such* a woman claims—all that I myself possess—if she will let me alone, and keep her distance from me. She may sing bass, but I do not wish to hear her. I believe in women; I believe they are the sweetest, purest, most unselfish, best part of the human race. They do sing the melody in all human life, as well as the melody in music." The more moderate duties of professional life might be assumed by women, but the most laborious matters would be left for the sterner sex. What would the people do with a child-bearing doctor? What an acceptable excuse for delay in night calls could be offered by the lactating doctor! The *Boston Medical and Surgical Journal* gives four reasons why the female is unadapted to the arduous duties of the medical profession:

*First*—A physiological obstacle, menstruation.

*Secondly*—That overflowing sympathy, which is such an attractive characteristic of woman, and which renders her a gentle and devoted nurse, stands in the way of her efficiency as a physician.

*Thirdly*—The peculiar characteristics of the female mind hardly afford scope for first-class attainments in either medical observation or practice.

*Fourthly*—That nicer sense of delicacy, which is the birthright of woman, and which we love to see her cherish, should interfere with her acquiring that knowledge of the human frame which is necessary to the medical practitioner, and oppose her familiar dealing with certain matters of daily medical experience. These reasonable objections are argued "*in extenso*" by the writer, and although exceptions may occur in females of a masculine cast of mind, let such be the exceptions, and the female apply her energies to feminine occupations, and not to the practice of medicine. We none of us would like to hear a woman sing bass, although she undoubtedly has perfect right to do so.

Another outgrowth of medicine is daily seen in our public prints in the form of advertisements of patent remedies and specifics for the cure of all diseases. These usually making great pretensions for the regard they have for the public health, while the advertiser and publisher are equally concerned to make a pecuniary gain to themselves the true basis of doing the public such wonderful good. No proprietor or publisher of a newspaper would admit many of these grossly immoral advertisements in their columns unless they were well paid; neither would the advertiser be able to pay them unless the immoral portion of our community contributed to his pecuniary needs. But public prints do pander to the follies of the people for the sake of money, for money seems to be the great leading aim and effort of those who control the public views. The following advertisement occurs in the same volume and number of one of our public newspapers, with one of the best written articles on criminal abortion that I have seen, viz:

"Dr. Clarke's celebrated Female Pills are a safe and effectual remedy in all cases. French Safes, $1 each. Address letters, Dr. CLARKE," etc.

In a later number of the same paper, we see *a* Dr. Bigelow advertising. He addresses as follows:

"YOUNG MEN, TAKE PARTICULAR NOTICE.—Dr. Bigelow devotes much of his time to the treatment of those cases caused by secret habits, which ruins both body and mind, unfitting the unfortunate individual for either business or society. The sad effect of these early habits or excess of riper years, is to weaken and debilitate the constitution, destroy the physical and mental powers, diminish and exhaust the vital energies of manhood; the pleasures of life are marred, *the object of marriage* frustrated, and existence itself rendered a term of unceasing misery and regret."

In the same advertisement he gives the following:

"N. B.—Ladies, send for a descriptive circular of Pessaric Remedies, the best preventive of conception known."

How much do such advertisers esteem public morality? What do they think concerning the object of marriage being frustrated, pleasures of life marred, or existence rendered miserable? They would give the public *brown bread pills* to restore the object of marriage, and strychnine to mend a miserable existence, *provided*, the public would give them money.

A writer from Paris, recently, says: "*Science* has been doing curious things here in Paris — seeking the physical causes of crime by comparing the viscera of honest and wicked men, looking into and dissecting dead hearts." Would that the people of America would examine the cause of crime by comparing the *viscera* of honest and wicked newspapers, looking into and dissecting the *dead hearts* of certain advertisers.

Medicine, although, a noble calling, honorable and worthy by those who make it such, is, as I have shown, not an exact science, and, for this reason, there have been outgrowths, monstrosities, fungi, thrown up in its path continually, and it is a part of our duty, as men who wish to elevate the standard of our profession, to beat down these enormities, by contributing from our abilities that which is honest, honorable and moral.

Faith is the Christian's anchor—the great starting point of the Christian's life—his hope of success—the steel-pointed staff with which he climbs the icy cliffs of difficulty! So medicine is the basis of a physician's outfit when he starts upon the life of toil to meet the giant disease as he devastates the domain. And this, like the Christian's anchor and staff, dropped at the proper time, taken with a firm hand and planted with its sharp point, will hold him well. Since Adam's fall, religion has been devising means of redemption. Since death commenced its devastation, physicians have been pitted against its progress, and, with what success, we all see. The fruit of the tree of knowledge is still eaten, and death still claims its victims by disease.

